# Convergent lines of evidence support *BIN1* as a risk gene of Alzheimer’s disease

**DOI:** 10.1186/s40246-021-00307-6

**Published:** 2021-01-30

**Authors:** Jin Zhu, Xia Liu, Hongtao Yin, Yan Gao, Hao Yu

**Affiliations:** 1 Department of Psychiatry, Jining Medical University, 133 He Hua Road, Jining, 272067 Shandong China; 2 Department of Psychiatry, Jining Psychiatric Hospital, Jining, 272051 Shandong China; 3 Department of Neurology, Zibo Central Hospital, 54 Gongqingtuan Xi Road, Zibo, 255036 China

**Keywords:** Alzheimer’s disease, Genome-wide association study (GWAS), Expression level, Candidate gene, Mendelian randomization

## Abstract

Genome-wide association studies (GWAS) have identified several susceptibility loci of Alzheimer’s disease (AD), which were mainly located in noncoding regions of the genome. Meanwhile, the putative biological mechanisms underlying AD susceptibility loci were still unclear. At present, identifying the functional variants of AD pathogenesis remains a major challenge. Herein, we first used summary data-based Mendelian randomization (SMR) with AD GWAS summary and expression quantitative trait loci (eQTL) data to identify variants who affects expression levels of nearby genes and contributed to the risk of AD. Using the SMR integrative analysis, we totally identified 14 SNPs significantly affected the expression level of 16 nearby genes in blood or brain tissues and contributed to the AD risk. Then, to confirm the results, we replicated the GWAS and eQTL results across multiple samples. Totally, four risk SNP (rs11682128, rs601945, rs3935067, and rs679515) were validated to be associated with AD and affected the expression level of nearby genes (*BIN1, HLA-DRA, EPHA1-AS1,* and *CR1*). Besides, our differential expression analysis showed that the *BIN1* gene was significantly downregulated in the hippocampus (*P* = 2.0 × 10^−3^) and survived after multiple comparisons. These convergent lines of evidence suggest that the *BIN1* gene identified by SMR has potential roles in the pathogenesis of AD. Further investigation of the roles of the *BIN1* gene in the pathogenesis of AD is warranted.

**Supplementary Information:**

The online version contains supplementary material available at 10.1186/s40246-021-00307-6.

## Introduction

Alzheimer’s disease (AD) is the most common neurodegenerative dementia and is clinically characterized by progressive loss of memory and deficits in thinking, problem solving, and language [1]. AD is highly heritable and its estimated heritability ranges from 60 to 80% [2]. Genome-wide association studies (GWAS) have identified multiple loci containing common variant risk alleles [3–5]. A large-scale GWAS of clinically diagnosed AD and AD-by-proxy (71,880 cases and 383,378 controls) identified 29 risk loci, involving 215 potential causative genes [6]. Another GWAS of late-onset Alzheimer’s disease (21,982 cases and 41,944 controls) identified five novel genome-wide loci, including *IQCK*, *ACE*, *ADAM10*, *ADAMTS1*, and *WWOX* [7]. These findings offer new routes to enhancing the diagnosis and the development of drug targets [8]. However, most of the identified risk single nucleotide polymorphisms (SNPs) are from noncoding regions [9, 10], making functional interpretation difficult.

One possible hypothesis is that the risk SNPs identified by GWAS contribute to the risk of diseases through affecting the expression level of nearby genes in different tissues [10, 11]. Consequently, to identify the functional variants from GWAS results, it is useful to integrate data of gene expression level (e.g., expression quantitative trait loci, eQTL) into GWAS data of diseases. Therefore, to prioritize candidate genes underlying GWAS hits, an integrated analysis method named summary data-based Mendelian randomization (SMR) was developed. Using the principles of Mendelian randomization, the SMR method could examine whether the expression level of a gene and a complex phenotype caused by pleiotropy and discern pleiotropy from linkage [12]. Through the SMR analysis, several novel candidate genes underlying GWAS hits of complex diseases or traits were prioritized for follow-up functional studies [13–16]. Strikingly, through integrating different omics data, we could gain further insights into the underlying genetic mechanisms of GWAS hits and disease [17].

To prioritize AD risk genes and investigate their roles in AD pathogenesis, we first combined the AD GWAS data and eQTL using SMR test. Then, we replicate the identified risk SNPs and genes across multiple samples. For the replicated risk genes, we compared the expression patterns in AD patients with healthy controls.

## Methods

### AD GWAS data

We obtained complete summary-level of AD GWAS from the website of Complex Trait Genomics lab (https://ctg.cncr.nl/software/summary_statistics). The AD GWAS consisted of 71,880 cases and 383,378 controls [6]. In PGC, IGAP, and ADSP consortia, individuals were of clinically diagnosed AD case-control status. The individuals with one or two parents diagnosed with AD in UKB were defined as proxy cases, and patients with two parents were upweighted. Meanwhile, participants with two parents without AD were defined as proxy controls, and older cognitively normal parents were also upweighted [6]. Recently, the value of by-proxy phenotypes has been demonstrated [5]. More details about demographic characteristics, genotyping, and statistical analysis were in the original study [6].

### eQTL data

In the SMR analysis, we integrated the AD GWAS data with brain and blood eQTL data, respectively. (1) For blood eQTL data (*n* = 31,684), the blood eQTL data was obtained from the eQTLGen consortium, which consisted of 31,684 individuals [18]. The associations between SNPs and gene expression levels were calculated using a Spearman correlation. In total, in the eQTLGen consortium, 19,960 genes that showed expression in the blood were tested and 238,340 *cis*-eQTL SNPs were identified. (2) For brain eQTL data (*n* = 1194), the brain eQTL study was from a meta-analysis of brain eQTL data [19]. To increase the power of detecting brain eQTLs, Qi et al. [19] performed a meta-analysis using three brain eQTL studies, including Genotype-Tissue Expression (GTEx) [20], CommonMind Consortium (CMC) [21], and the Religious Orders Study and the Rush Memory and Aging Project (ROSMAP) [17]. To correct the overlapped sample, the MeCS approach was used to combine the eQTL results of 10 brain regions of GTEx database [19]. In the present study, we only used the SNPs within 1 Mb distance from each gene. More details were in the original paper [18, 19].

### SMR analysis

To prioritize candidate causal genes of AD, we integrated GWAS and eQTL data through SMR method, which examine the putative pleiotropic relationships between AD and eQTL [12]. The SMR method mainly comprises of two steps. First, genetic variations are used as instrumental variables to examine for causative effect of gene expression on AD. Second, we applied the heterogeneity in dependent instruments (HEIDI) test implemented in SMR software to distinguish the causality and pleiotropy model from the linkage model. If the HEIDI test is significant (*P*_HEIDI_ < 0.05), the identified genes by SMR can be a result of linkage. To account for multiple testing, we adjusted *P*_SMR_ values using the Bonferroni approach. The set associated genes were defined as genes with a Bonferroni-corrected *P*_SMR_ < 0.05 and *P*_HEIDI_ > 0.05. The SMR software was downloaded from https://cnsgenomics.com/software/smr.

### AD GWAS data for replication analysis

To further replicate the AD GWAS results in SMR, we investigated the associations between the identified risk SNPs and AD using the GWAS summary data of International Genomics of Alzheimer’s Project (IGAP), which is a large three-stage study based upon genome-wide association studies (GWAS) on individuals of European ancestry [22]. In our study, we extracted the association results from the stage 1 results of IGAP, consisting of 21,982 AD cases and 41,944 normal controls [22]. More details of samples, quality control, imputation, and statistical analysis were in the original study [22].

### eQTL data for replication analysis

To validate the eQTL results in SMR, we examined the *cis*-eQTL effects of risk SNPs using two public databases as follows. First, we examined the blood eQTL results using the GTEx database. The genotype data used for eQTL analyses in GTEx was based on whole exome sequencing from 838 donors, which all had RNA-seq data available [23]. The associations between was performed using FastQTL. Totally, 49 tissues were tested in GTEx. Second, in the PsychENCODE database, to replicate the brain eQTL results of SMR analysis, we used the cis-eQTL data in the prefrontal cortex from the PsychENCODE project (*n* = 1387) [24]. The eQTL analyses of PsychENCODE were performed including100 hidden covariate factors as covariates. Only the data of SNPs in a 1-Mb window around each gene are available.

### Differential expression analysis of risk genes

To compare the expression level of the risk genes in AD cases with healthy controls, we performed the differential expression analysis using the comprehensive AlzData database (http://www.alzdata.org/) [25]. The AlzData database consisted of the expression data of four brain regions, including entorhinal cortex (EC), hippocampus (HIPP), temporal cortex (TC), and frontal cortex (FC). After conducting the cross-platform normalization, the normalized expression data sets were used to perform different expression analysis between AD cases and controls, using the linear regression model implemented in R package limma [25]. We used the false discovery rate (FDR) method to correct for multiple comparisons [25].

## Results

### SMR analysis identified risk variants and genes for AD

**Fig. 1:**
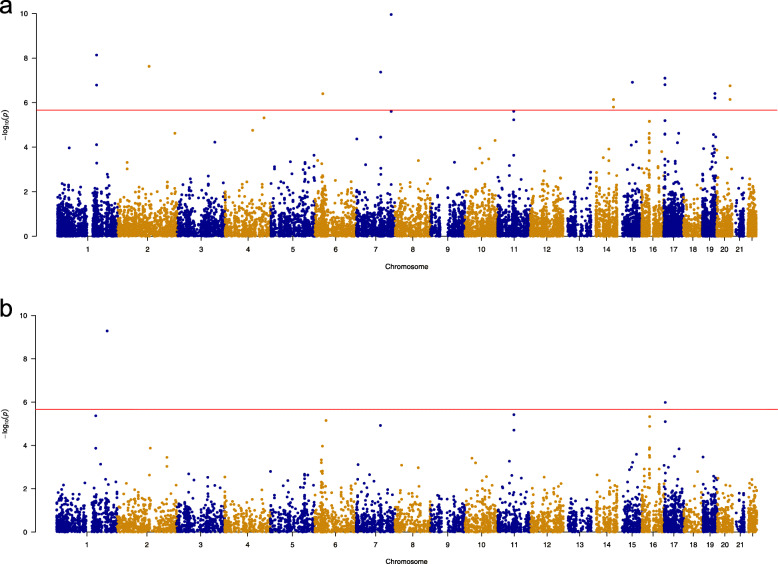
Manhattan plots of SMR results. **a** SMR analysis using the AD GWAS and blood eQTL results. **b** SMR analysis using the AD GWAS and brain eQTL results. The *y* axis shows the − log10 (*P* values) of SMR tests. The red line represent the significant level (*P* < 2.17e− 6)

To identify functional variants related to AD, we conducted SMR analysis using the genome-wide significantly associated genetic variants as an instrumental variable to examine the association between the expression level of each gene and AD. In the SMR analysis, we integrated AD GWAS with eQTL data from the blood and brain, respectively. Totally, 6 genes in the brain and 22 genes in blood were identified after correcting for multiple comparisons (*P*_SMR_ < 0.05/*n*; *n* = 23048; *n* represent the number of tests across blood and brain SMR analysis; Fig. 1 and Table 1). Then, we performed the HEIDI analysis for the identified genes to reduce the effect of potential linkage. Of the genes identified in the SMR analysis, 2 genes in the brain and 15 genes in blood were survived after the HEIDI test (*P*_HEIDI_ > 1.79 × 10^−3^, i.e., 0.05/*n*, with *n* = 28 being the total number of HEIDI tests) (Fig. 1 and Table 1), with one gene in common and 16 unique genes in total. Besides, the SMR analysis identified 14 AD risk SNPs (Fig. 1 and Table 1).

**Table 1:** Integrative analysis of GWAS and eQTL identified risk genes of AD

Chr	BP	SNP	A1	A2	Gene	b_GWAS	se_GWAS	p_GWAS	b_eQTL	se_eQTL	p_eQTL	b_SMR	se_SMR	p_SMR	p_HEIDI
**Integrative analysis of AD GWAS and blood eQTL**															
1	161156033	rs11585858	A	C	*B4GALT3*	0.016	0.003	5.58E− 10	− 0.168	0.011	1.07E− 57	− 0.093	0.016	7.33E− 09	3.33E− 03
1	161186313	rs4379692	T	C	*NDUFS2*	0.012	0.002	8.25E− 08	− 0.231	0.009	4.34E− 132	− 0.053	0.010	1.63E− 07	1.22E− 01
2	127839474	rs11682128	A	G	*BIN1*	0.031	0.006	2.21E− 08	0.742	0.009	0.00E+ 00	0.042	0.008	2.36E− 08	2.12E− 03
6	32573415	rs601945	G	A	*HLA-DRA*	− 0.019	0.003	1.38E− 10	− 0.117	0.014	1.39E− 16	0.160	0.032	3.99E− 07	4.25E− 03
7	99803412	rs2950517	G	C	*CASTOR3*	− 0.014	0.002	2.15E− 09	0.124	0.009	3.30E− 42	− 0.114	0.021	4.26E− 08	8.74E− 03
7	143104331	rs3935067	C	G	*EPHA1-AS1*	0.015	0.002	6.52E− 11	− 0.466	0.011	0.00E+ 00	− 0.031	0.005	1.11E− 10	6.94E− 03
14	92955385	rs17783630	C	A	*SLC24A4*	0.011	0.002	6.71E− 07	− 0.503	0.008	0.00E+ 00	− 0.021	0.004	7.31E− 07	3.28E− 02
14	92955385	rs17783630	C	A	*RIN3*	0.011	0.002	6.71E− 07	− 0.165	0.009	9.12E− 77	− 0.064	0.013	1.58E− 06	6.15E− 02
15	63571820	rs75763893	T	C	*APH1B*	0.017	0.003	9.15E− 08	0.681	0.018	0.00E+ 00	0.025	0.005	1.22E− 07	5.00E− 02
17	5014212	rs73976310	A	G	*AC012146.7*	0.018	0.003	7.04E− 08	− 0.743	0.013	0.00E+ 00	− 0.024	0.004	8.00E− 08	1.55E− 02
17	5014212	rs73976310	A	G	*ZNF232*	0.018	0.003	7.04E− 08	− 0.305	0.013	8.16E− 114	− 0.058	0.011	1.57E− 07	7.88E− 02
19	51731176	rs7245846	A	G	*SIGLEC22P*	− 0.012	0.002	1.19E− 07	0.166	0.009	2.38E− 70	− 0.072	0.014	3.91E− 07	1.05E− 01
19	51726911	rs1710398	C	A	*CD33*	0.011	0.002	4.46E− 07	0.290	0.009	3.60E− 221	0.037	0.007	6.18E− 07	4.27E− 03
20	54989833	rs6014722	A	T	*CASS4*	− 0.022	0.004	2.62E− 09	− 0.141	0.016	3.81E− 19	0.159	0.032	7.20E− 07	4.18E− 03
20	54987216	rs17462136	C	G	*RPL39P*	− 0.022	0.004	6.43E− 09	− 0.208	0.017	5.07E− 33	0.106	0.020	1.76E− 07	2.50E− 03
**Integrative analysis of AD GWAS and brain eQTL**															
1	207750568	rs679515	C	T	*CR1*	− 0.025	0.003	1.10E− 18	− 0.587	0.067	2.10E− 18	0.042	0.007	5.17E− 10	4.67E− 02
17	5014212	rs73976310	G	A	*AC012146.7*	− 0.018	0.003	7.04E− 08	0.721	0.062	6.19E− 31	− 0.025	0.005	1.03E− 06	2.73E− 01

We used the blood eQTL results of eQTLGen consortium [18], and the brain eQTL data from the study by Qi et al. [19]. *Chr* chromosome, *BP* base position, *Gene* gene symbol, *A1* reference allele, *A2* alternative allele, *b_GWAS* regression coefficient for AD (positive value indicates increased risk for AD in carriers of the alternative allele and negative value represents decreased risk for AD), *se_GWAS* standard error in GWAS, *p_GWAS p* value of GWAS, *b_eQTL* beta value of eQTL (positive values indicate increased expression with alternative allele and negative value represents decreased expression level), *se_eQTL* standard error of eQTL, *p_eQTL p* value of eQTL, *b_SMR* beta value of SMR test (positive values indicate increased expression level of target gene contributed to the risk of AD and negative values represent the opposite results), *se_SMR* standard error of SMR test, *p_SMR p* value of SMR test

### Replication analysis of GWAS and eQTL results

**Fig. 2:**
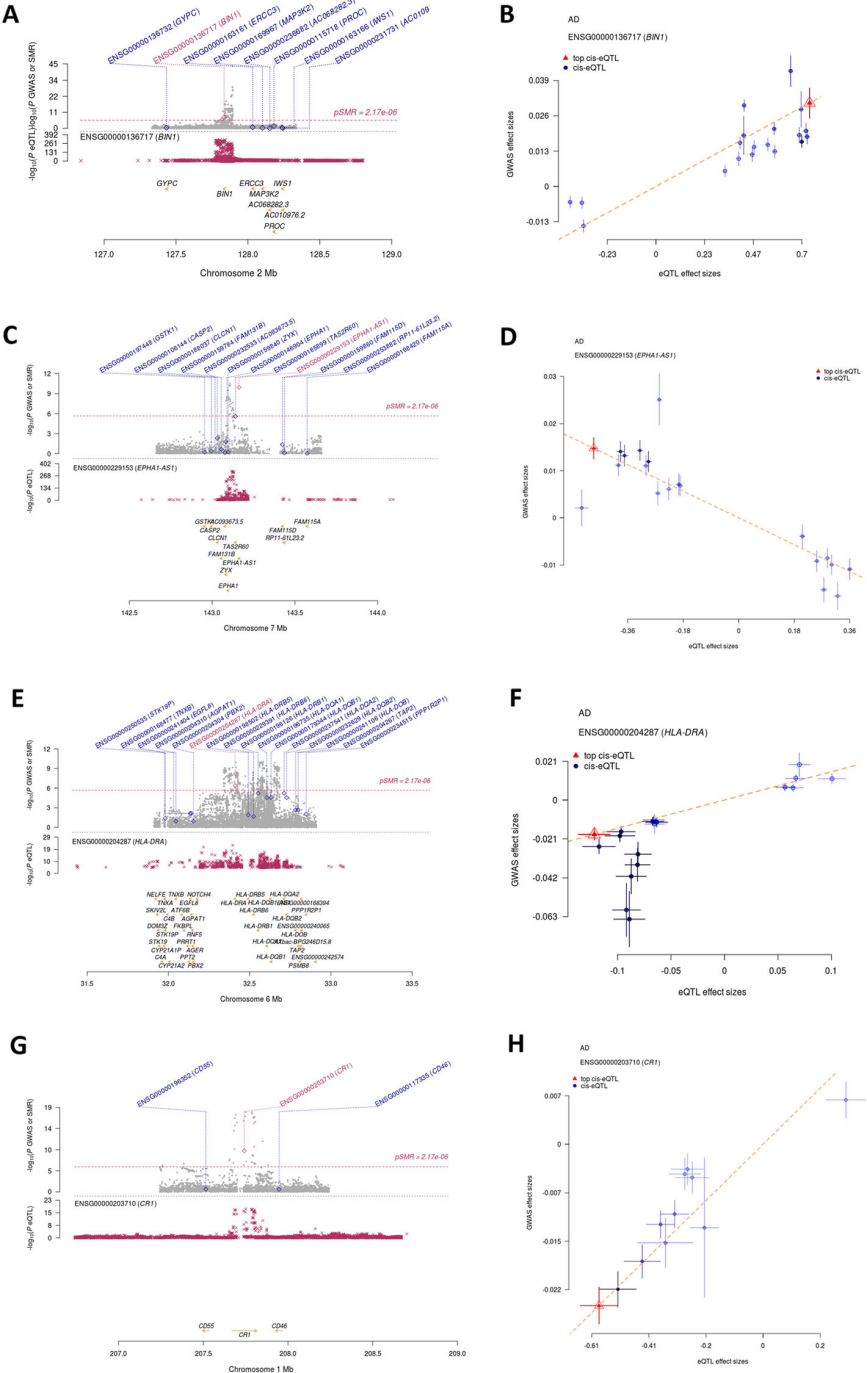
Prioritizing genes at four loci for AD. **a**, **c**, **e**, **g** The brown dots at top plot represent the association between SNPs and AD in GWAS, diamonds represent the *P* values of SMR analysis, and triangles stand for genes without a *P*_eQTL_ < 5.0 × 10^−8^. In the bottom plot, the SNPs with *P*_eQTL_ of eQTL study were plotted. The genes that survived after the SMR and HEIDI tests were highlighted using red color. **b**, **d**, **f**, **h** We showed the effect estimates of SNPs from AD GWAS plotted against those for SNPs from the eQTL analysis. The orange lines represent the estimate of effect size at the top cis-eQTL. Error bars represent the standard errors of SNP effects size

To further investigate the associations between 14 risk SNPs and AD, we replicated the SNPs results using a meta-analysis of AD GWASs. All the 14 SNPs showed nominally significant association with AD (*P* < 0.05) with the same effect directions in IGAP GWAS dataset (Supplementary Table 1). Nine risk SNPs were still significant after Bonferroni correction (*P* < 0.05/14 = 3.57 × 10^−3^; Supplementary Table 1). To further examine whether the 9 SNPs were associated with the expression level of nearby genes, we replicated the blood and brain eQTL effects identified by SMR using GTEx and PsychoENCODE datasets, respectively. Of the eight blood eQTL effects identified by SMR, 3 SNPs (rs11682128, rs601945, and rs3935067) showed genome-wide *cis*-eQTL effects in blood tissues (*P* < 5 × 10^−8^; Supplementary Table 2). In the replication analysis of brain eQTL effects, the SNP rs679515 was replicated in PsychoENCODE database (Supplementary Table 3). Totally, 4 SNPs (rs11682128, rs601945, rs3935067, and rs679515) are replicated in blood and brain eQTL databases, respectively. Therefore, four SNP-gene combinations, rs11682128-*BIN1*, rs601945-*HLA-DRA*, rs3935067-*EPHA1-AS1*, and rs679515-*CR1*, were strongly suggested to be promising candidates for AD risk. To better view the SMR results of these 4 SNPs, we plotted the GWAS, eQTL results, and SMR results in Fig. 2.

### Differential expression analysis of the AD risk genes

**Fig. 3:**
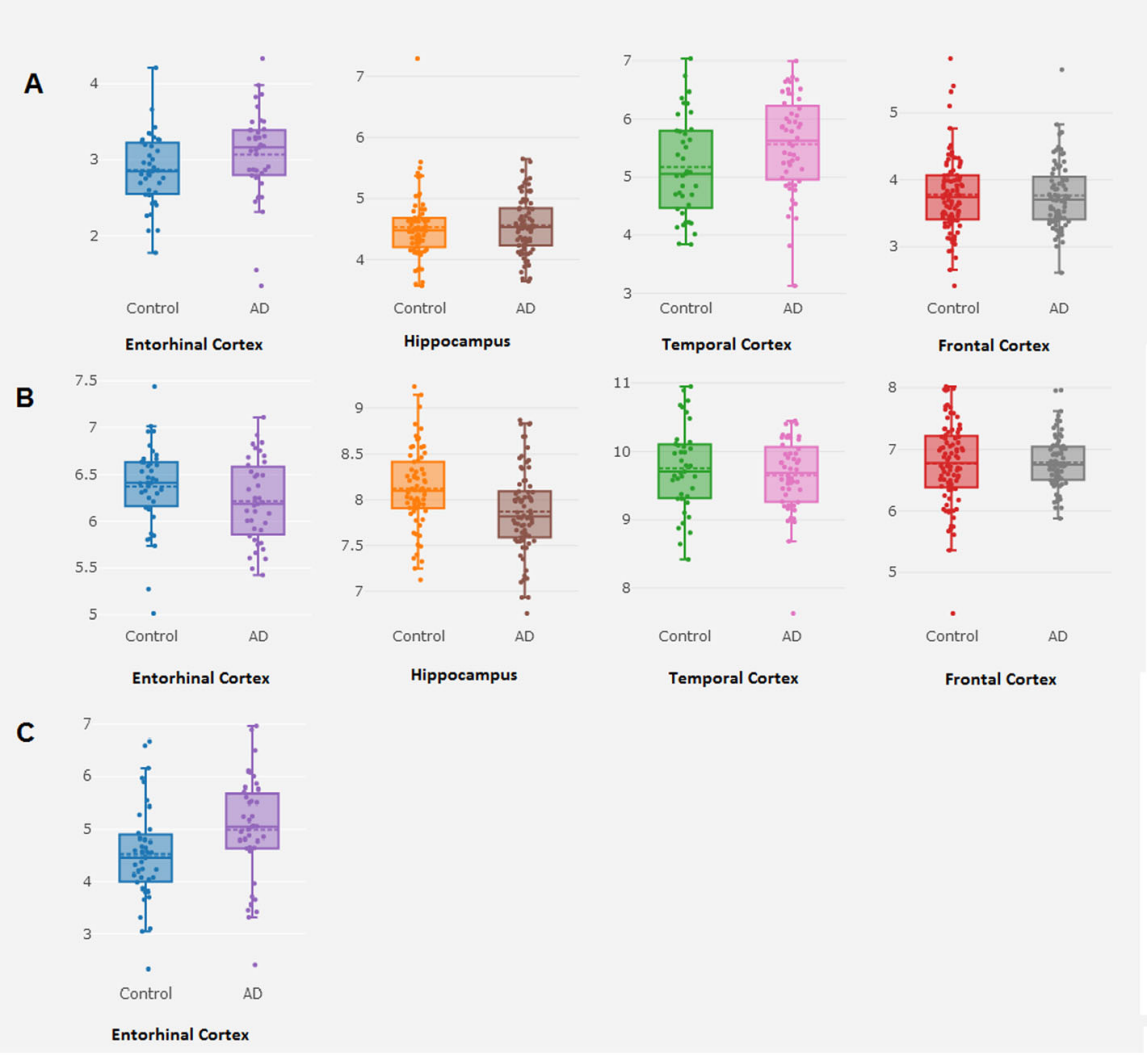
Differential expression analysis of candidate genes using AlzData database. We examined the expression level of the risk genes in AD cases and healthy controls using the AlzData database (http://www.alzdata.org/). **a** Differential expression analysis of *CR1*. **b** Differential expression analysis of *BIN1.* **c** Differential expression analysis of *HLA-DRA*

Considering that the expression level of risk genes might change and contribute to AD risk, we further investigated whether the four risk genes are differentially expressed in AD patients compared to controls by using the AlzData database (http://www.alzdata.org/) [25]. Comparing AD patients with controls, the *BIN1* gene was significantly downregulated in the hippocampus (*P* = 0.002; Fig. 3 and Table 2), surviving after FDR correction in the original study [25]. Based on the SMR results, our differential expression analysis further supports *BIN1* as an AD risk gene. However, the *HLA-DRA* and *CR1* genes showed no significant differential expression pattern between AD cases and controls (Fig. 3 and Table 2). The gene *EPHA1-AS1* was not available in the AlzData database.

**Table 2:** Expression analysis of risk genes in AD cases and controls using AlzData database

Gene	Entorhinal cortex			Hippocampus			Temporal cortex			Frontal cortex		
	FC	***P***	FDR	FC	***P***	FDR	FC	***P***	FDR	FC	***P***	FDR
CR1	0.23	0.071	0.197	− 0.01	0.896	0.954	0.39	0.033	0.118	− 0.04	0.655	0.906
BIN1	− 0.12	0.265	0.452	− 0.26	0.002	0.028	− 0.09	0.458	0.67	0.11	0.13	0.268
HLA-DRA	0.46	0.042	0.142	NA	NA	NA	NA	NA	NA	NA	NA	NA

We investigated whether the identified risk genes are differentially expressed in AD patients compared to controls, using a comprehensive database AlzData (http://www.alzdata.org/). *FC* fold change, *FDR* false discovery rate, *NA* not available

## Discussion

Recently, hundreds of AD risk SNPs have been identified in GWAS [3–5]. The large majority of risk loci of AD are located in noncoding regions of the genome. How to identify the genetic mechanisms underlying risk SNPs remains a major challenge. Moreover, given that the gene density and linkage disequilibrium structure, it is difficult to identify causal SNPs for AD. Based on GWAS results alone, we could not predict whether the risk SNPs have functional consequences. In this study, by using the SMR analysis, we systematically integrate the AD GWAS and blood or brain eQTL data. Ultimately, we identified 14 risk SNPs, which affected the expression level of 16 nearby genes and contributed to risk for AD. Our results support that the gene expression might play a mediating role for effects at these risk SNPs. Our findings not only confirmed previous findings, but also highlighted new risk SNPs and genes underlying AD. Through SMR analysis, we identified eight novel risk SNPs that were not genome-wide significant in the original AD GWAS [6]. Hence, some missing heritability might be identified using SMR. To further confirm the SMR results, we replicated the GWAS and eQTL results. Totally, four genes (*BIN1*, *HLA-DRA*, *EPHA1-AS1*, and *CR1*) were strongly suggested to be promising candidates for AD risk. We expect these SNPs to be detected in future genetic association studies with larger sample sizes. Then, we conducted the differential expression analysis to compare the expression level of four replicated genes in AD cases and controls. Only the *BIN1* gene showed significant differential expression level. Therefore, we demonstrated that the *BIN1* gene contributed to the risk of AD.

Our study provides convergent lines of evidence supporting the *BIN1* gene as a candidate gene of AD. First, we identified the AD risk gene *BIN1* by integrating large-scale GWAS and eQTL with SMR analysis. Second, the SMR results were replicated across GWAS and eQTL databases. Third, given that the SMR test identifies AD-associated genes with the underlying assumption that expression levels of those genes may have a role in AD pathogenesis, we explored whether AD risk genes identified by SMR were differentially expressed in AD patients compared to controls, using the comprehensive AlzData database [25]. Comparing AD patients with controls, the *BIN1* gene was also significantly downregulated in the hippocampus. However, there were no significant differences in the expression of other genes. This might be due to the lack of power and heterogeneity of different expression data sets.

Our SMR results identified that risk SNPs caused the dysregulation of the gene expression level and increased the risk for AD. However, our findings for an association between *BIN1* and risk of AD are mixed, suggesting the complex role of *BIN1* in AD risk. First, our SMR results in blood are consistent with previous studies. At the *BIN1* locus, our SMR results suggested that the risk allele A of SNP rs11682128 could upregulate the expression level of the *BIN1* gene in blood and increase the AD risk. Consistent with our results, higher *BIN1* mRNA levels in blood were detected in AD patients compared with controls [26]. Next, our results of the expression level of *BIN1* in brain were different from previous findings. Using AlzData database [25], we found that the *BIN1* gene was significantly downregulated in AD patients compared to controls in hippocampus (Table 2). Coincidentally, the AD risk allele of *BIN1* showed significant associations with memory deficits, hippocampal volume, and functional connectivity, suggesting the potential role of *BIN1* in AD pathogenesis [27, 28]. However, most of previous evidence showed an increase of *BIN1* expression level in the brains of patients with AD [29, 30]. Moreover, the increased *BIN1* expression level has also been linked to tau pathology [29–32]. These inconsistent findings might be interpreted by the different functions of different domains in *BIN1* gene. Compared to healthy controls, the amount of the largest isoform of *BIN1* was found to be significantly reduced in the AD brain, and smaller *BIN1* isoforms were significantly increased [31]. Third, we found inconsistency between SMR results in blood and differential expression results in brain. This phenomenon may be caused by diverse roles of *BIN1* in AD pathology. Many kinds of evidence has shown that *BIN1* may involve in several AD-related pathways in AD, including tau and amyloid pathology, and relevant pathways such as inflammation, apoptosis, and calcium homeostasis [33]. Additionally, though previous studies suggested that the genetic architecture underlies the regulation of gene expression across tissues, there are still some genetic differences between tissues [19]. Therefore, we inferred that the different functions of different domains and distinct tissue localizations may indicate the role of *BIN1* in the pathogenesis of AD. However, adequate and reliable research on *BIN1* in AD is still needed in the future.

Compared with these two previous studies, our present study has some similarities and differences. Previous studies have demonstrated that the SMR method was helpful to prioritize novel AD-associated genes. For example, Hu et al. identified several candidate genes by integrating two AD GWASs and five eQTL studies using SMR test [34]. Then, to improve their result, Zhao et al. performed a meta-analysis using five AD GWAS and integrated the meta results with eQTL using SMR [35]. Several risk genes were identified to be associated with AD in expression levels by pleiotropy [35]. Notably, all three studies applied SMR to AD GWAS and brain eQTL data. Hu et al. used two AD GWAS (25,580 AD cases and 48,466 controls) and five eQTL to perform SMR test [34]. Zhao et al. used summary statistics from a mega-analysis of five GWAS datasets (369,957 participants) and three brain eQTL [35]. Meanwhile, our present study used GWAS data (71,880 AD cases and 383,378 controls) from the mega-analysis by Jansen et al. [6], blood eQTL data (*n* = 31,684), and brain eQTL data (*n* = 1194). Generally, the current study had increased the sample size compared with previous studies [34, 35] and then might improve the statistical power and accuracy of SMR statistical results. The current study identified several risk genes which were not identified by two previous SMR studies [34, 35], such as *NDUFS2, CASTOR3, APH1B,* and *B4GALT3,* extending the findings of previous studies. Second, we not only prioritized risk gene using SMR test, but also replicated the SMR results in IGAP GWAS, GTEx, and PsychoENCODE databases. Besides, we also explored the functional roles of these identified SNPs using differential gene expression patterns in AD patients and controls. These identified genes using the integrated computational analyses could be prioritized based on biological relevance using follow-up laboratory-based validation using in vitro and in vivo model systems.

Our study has a number of limitations. First, in the first-stage of SMR analysis, some AD cases of the GWAS sample were defined based on the parental diagnoses. Therefore, the SNP associations might be biased. However, the strategy of AD-by-proxy was demonstrated to be robust. For example, the diagnosed case-control status and the UKB by-proxy phenotype showed high genetic correlation, and a large proportion of novel loci were replicated in the independent cohort [6]. Furthermore, we replicated the GWAS results using IGAP samples, which were clinically diagnosed. Therefore, the biases in AD associations caused by misdiagnosis might be relatively modest. Second, our study provides several lines of evidence that the *BIN1* gene contributes to the risk of AD. However, the potential casual gene *BIN1* was identified through using the GWAS and eQTL results of European population. These prioritized genes might not be associated with AD in other populations. Thus, these results should be validated in other populations.

## Conclusions

In this study, we combined the GWAS and eQTL datasets and identified the risk SNP rs11682128, which might contribute to AD risk through affecting the expression level of *BIN1* gene. Our SMR analysis could not only identify functional genes but improve our understanding of the pathogenesis mechanism underlying AD.

## Supplementary Information

**Additional file 1: Supplementary Table 1.** Replication analysis for the association between risk SNPs and AD. **Supplementary Table 2.** Replication analysis for the blood eQTL results in the GTEx database. **Supplementary Table 3.** Replication analysis for the brain eQTL results in the PsychENCODE database.

## Data Availability

Not applicable
