# Supplementary material for: Convergent lines of evidence support BIN1 as a risk gene of Alzheimer’s disease
Source: Hum Genomics. 2021 Jan 30;15:9. doi: 10.1186/s40246-021-00307-6 (PMC7847034; doi:10.1186/s40246-021-00307-6)
Supplement: Supplementary file 1 — Additional file 1: Supplementary Table 1. Replication analysis for the association between risk SNPs and AD. Supplementary Table 2. Replication analysis for the blood eQTL results in the GTEx database. Supplementary Table 3. Replication analysis for the brain eQTL results in the PsychENCODE database. [file 40246_2021_307_MOESM1_ESM.docx]

**Supplementary Table 1. Replication analysis for the association between risk SNPs and AD.**

| **CHR** | **BP** | **SNP** | **A1** | **A2** | **Gene** | **BETA** | **SE** | **P** |
| --- | --- | --- | --- | --- | --- | --- | --- | --- |
| 1 | 161156033 | rs11585858 | A | C | B4GALT3 | 0.037 | 0.017 | 2.73E-02 |
| 2 | 127839474 | rs11682128 | A | G | BIN1 | 0.065 | 0.015 | 1.47E-05 |
| 19 | 51726911 | rs1710398 | A | C | CD33 | -0.057 | 0.015 | 1.03E-04 |
| 20 | 54987216 | rs17462136 | C | G | RPL39P | -0.123 | 0.026 | 2.47E-06 |
| 14 | 92955385 | rs17783630 | A | C | SLC24A4 | -0.041 | 0.015 | 6.11E-03 |
| 7 | 99803412 | rs2950517 | C | G | CASTOR3 | 0.041 | 0.016 | 1.11E-02 |
| 7 | 143104331 | rs3935067 | C | G | EPHA1-AS1 | 0.063 | 0.015 | 3.92E-05 |
| 1 | 161186313 | rs4379692 | T | C | NDUFS2 | 0.033 | 0.015 | 3.02E-02 |
| 20 | 54989833 | rs6014722 | A | T | CASS4 | -0.124 | 0.026 | 2.17E-06 |
| 6 | 32573415 | rs601945 | A | G | HLA-DRA | 0.085 | 0.021 | 6.53E-05 |
| 1 | 207750568 | rs679515 | T | C | CR1 | 0.151 | 0.018 | 1.56E-16 |
| 19 | 51731176 | rs7245846 | A | G | SIGLEC22P | -0.065 | 0.016 | 3.93E-05 |
| 17 | 5014212 | rs73976310 | A | G | LOC101928000 | 0.054 | 0.023 | 2.14E-02 |
| 15 | 63571820 | rs75763893 | T | C | APH1B | 0.089 | 0.026 | 5.80E-04 |

Chr., chromosome; BP, bp position of the SNP; OR, odds ratio; SNP, single-nucleotide polymorphism. All positions are relative to UCSC hg19. A1/A2, reference and alternate allele.

**Supplementary Table 2. Replication analysis for the blood eQTL results in the GTEx database.**

| **SNP** | **CHR** | **BP** | **A1** | **A2** | **Gene** | **Beta** | **SE** | **P** |
| --- | --- | --- | --- | --- | --- | --- | --- | --- |
| rs11682128 | 2 | 127839474 | A | G | BIN1 | 0.229 | 0.023 | 3.11E-23 |
| rs601945 | 6 | 32573415 | G | A | HLA-DRB6 | 0.505 | 0.078 | 1.01E-10 |
| rs601945 | 6 | 32573415 | G | A | HLA-DRB1 | -0.233 | 0.031 | 9.64E-14 |
| rs601945 | 6 | 32573415 | G | A | HLA-DQB1 | -0.394 | 0.066 | 2.21E-09 |
| rs601945 | 6 | 32573415 | G | A | HLA-DQA2 | 1.058 | 0.083 | 3.74E-37 |
| rs601945 | 6 | 32573415 | G | A | HLA-DQB2 | 0.534 | 0.081 | 5.41E-11 |
| rs3935067 | 7 | 143104331 | C | G | TAS2R60 | -0.390 | 0.067 | 6.76E-09 |
| rs3935067 | 7 | 143104331 | C | G | EPHA1-AS1 | -0.717 | 0.076 | 2.94E-21 |
| rs73976310 | 17 | 5014212 | A | G | AC012146.7 | -0.488 | 0.087 | 1.80E-08 |

Chr, Chromosome; Gene, Gene name; BP, bp position of the SNP; A1, SNP reference allele; A2, SNP alternative allele; beta, regresion coefficient of expression level on SNP; se, standard error; p, p-value for beta different from zero.

**Supplementary Table 3. Replication analysis for the brain eQTL results in the PsychENCODE database.**

| **SNP** | **Chr** | **BP** | **A1** | **A2** | **Gene** | **b** | **SE** | **p** |
| --- | --- | --- | --- | --- | --- | --- | --- | --- |
| rs679515 | 1 | 207750568 | C | T | CR1 | -0.833 | 0.054 | 2.94E-53 |
| rs73976310 | 17 | 5014212 | A | G | ZNF232 | -0.584 | 0.058 | 3.19E-24 |
| rs73976310 | 17 | 5014212 | A | G | AC012146.7 | -0.937 | 0.056 | 1.05E-63 |
| rs73976310 | 17 | 5014212 | A | G | NUP88 | -0.405 | 0.058 | 3.34E-12 |
| rs6014722 | 20 | 54989833 | A | T | CASS4 | 0.373 | 0.059 | 3.02E-10 |
| rs2950517 | 7 | 99803412 | C | G | PVRIG | -0.353 | 0.043 | 2.66E-16 |

Chr, Chromosome; Gene, Gene name; BP, bp position of the SNP; A1, SNP reference allele; A2, SNP alternative allele; beta, regresion coefficient of expression level on SNP; se, standard error; p, p-value for beta different from zero.
